# 3D printing of conducting polymers

**DOI:** 10.1038/s41467-020-15316-7

**Published:** 2020-03-30

**Authors:** Hyunwoo Yuk, Baoyang Lu, Shen Lin, Kai Qu, Jingkun Xu, Jianhong Luo, Xuanhe Zhao

**Affiliations:** 1 Department of Mechanical Engineering, Massachusetts Institute of Technology, Cambridge, MA 02139 USA; 2 Flexible Electronics Innovation Institute, Jiangxi Science and Technology Normal University, Nanchang, 330013 China; 3 School of Pharmacy, Jiangxi Science and Technology Normal University, Nanchang, 330013 China; 4 Department of Civil and Environmental Engineering, Massachusetts Institute of Technology, Cambridge, 02139 USA; 5 Department of Neurobiology, Key Laboratory of Medical Neurobiology of the Ministry of Health of China, Collaborative Innovation Center for Brain Science, Zhejiang University of Medicine, Hangzhou, Zhejiang, 310058 China

**Keywords:** Mechanical engineering, Electronic devices, Gels and hydrogels

## Abstract

Conducting polymers are promising material candidates in diverse applications including energy storage, flexible electronics, and bioelectronics. However, the fabrication of conducting polymers has mostly relied on conventional approaches such as ink-jet printing, screen printing, and electron-beam lithography, whose limitations have hampered rapid innovations and broad applications of conducting polymers. Here we introduce a high-performance 3D printable conducting polymer ink based on poly(3,4-ethylenedioxythiophene):polystyrene sulfonate (PEDOT:PSS) for 3D printing of conducting polymers. The resultant superior printability enables facile fabrication of conducting polymers into high resolution and high aspect ratio microstructures, which can be integrated with other materials such as insulating elastomers via multi-material 3D printing. The 3D-printed conducting polymers can also be converted into highly conductive and soft hydrogel microstructures. We further demonstrate fast and streamlined fabrications of various conducting polymer devices, such as a soft neural probe capable of in vivo single-unit recording.

## Abstract (web summary)

Conducting polymers are promising materials for diverse applications but the fabrication of conducting polymers mostly relies on conventional fabrication techniques. Here the authors introduce a high performance 3D printable conducting polymer ink to take full advantage of advanced 3D printing.

## Introduction

Conducting polymers, a class of polymers with intrinsic electrical conductivity, have been one of the most promising materials in applications as diverse as energy storage^1^, flexible electronics^2^, and bioelectronics^3^, owing to their unique polymeric nature as well as favorable electrical and mechanical properties, stability, and biocompatibility. Despite recent advances in conducting polymers and their applications, the fabrication of conducting polymer structures and devices have mostly relied on conventional manufacturing techniques such as ink-jet printing^4–6^, screen printing^7^, aerosol printing^8–10^, electrochemical patterning^11–13^, and lithography^14–16^ with limitations and challenges. For example, these existing manufacturing techniques for conducting polymers are limited to low-resolution (e.g., over 100 µm), two-dimensional (e.g., low aspect ratio) patterns, and/or complex and high cost procedures (e.g., multi-step processes in clean room involving alignments, masks, etchings, post-assemblies)^4,5,7,14–16^ (Supplementary Table 1), which have hampered rapid innovations and broad applications of conducting polymers. Unlike these conventional approaches, three-dimensional (3D) printing offers capabilities to fabricate microscale structures in a programmable, facile, and flexible manner with a freedom of design in 3D space^17,18^ (Supplementary Table 1). For example, recent developments of 3D printable materials such as metals^19,20^, liquid metals^21^, hydrogels^22,23^, cell-laden bioinks^24–26^, glass^27^, liquid crystal polymers^28^, and ferromagnetic elastomers^29^ have greatly expanded the accessible materials library for 3D printing. While intensive efforts have been devoted to 3D printing of conducting polymers, only simple structures such as isolated fibers have been achieved^30–32^ owing to insufficient 3D printability of existing conducting polymer inks.

Here we invent a high-performance 3D printable ink based on one of the most widely utilized conducting polymers poly(3,4-ethylenedioxythiophene):polystyrene sulfonate (PEDOT:PSS) to take advantage of advanced 3D printing for the fabrication of conducting polymers. To achieve favorable rheological properties for 3D printing, we develop a paste-like conducting polymer ink based on cryogenic freezing of aqueous PEDOT:PSS solution followed by lyophilization and controlled re-dispersion in water and dimethyl sulfoxide (DMSO) mixture. The resultant conducting polymer ink exhibits superior 3D printability capable of high resolution (over 30 µm), high aspect ratio (over 20 layers), and highly reproducible fabrication of conducting polymers, which are also readily integratable with other 3D printable materials such as insulating elastomers by multi-material 3D printing. Dry-annealing of the 3D-printed conducting polymers provides highly conductive (electrical conductivity over 155 S cm^−1^) and flexible PEDOT:PSS 3D microstructures in the dry state. Moreover, the dry-annealed 3D-printed conducting polymers can be readily converted into a soft (Young’s modulus below 1.1 MPa) yet highly conductive (electrical conductivity up to 28 S cm^−1^) PEDOT:PSS hydrogel via subsequent swelling in the wet environment. We further demonstrate a facile and streamlined fabrication of various functional conducting polymer devices by multi-material 3D printing, including a high-density flexible electronic circuit and a soft neural probe capable of in vivo single-unit recording.

## Results

### 3D printable conducting polymer ink

**Fig. 1:**
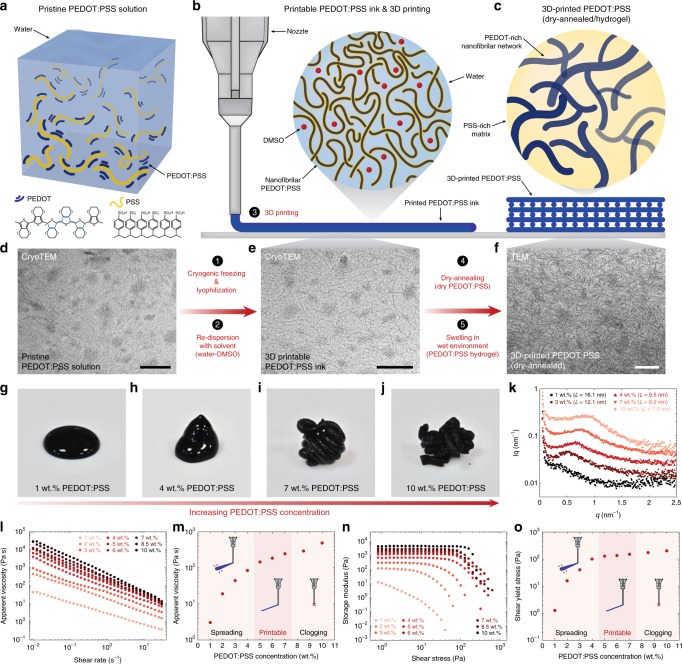
Design of 3D printable conducting polymer ink. **a**, **b**, Pristine PEDOT:PSS solution (**a**) can be converted into a 3D printable conducting polymer ink (**b**) by lyophilization in cryogenic condition and re-dispersion with a solvent. **c**, 3D-printed conducting polymers can be converted into a pure PEDOT:PSS both in dry and hydrogel states by dry-annealing and subsequent swelling in wet environment, respectively. **d** CryoTEM image of a pristine PEDOT:PSS solution. **e** CryoTEM image of a 3D printable conducting polymer ink. **f** TEM image of a dry-annealed 3D-printed conducting polymer. **g**–**j** Images of re-dispersed suspensions with varying PEDOT:PSS nanofibril concentration. **k** SAXS characterization of conducting polymer inks with varying PEDOT:PSS nanofibril concentration. The d-spacing *L* is calculated by the Bragg expression *L* = 2π/*q*_max_. **l** Apparent viscosity as a function of shear rate for conducting polymer inks of varying PEDOT:PSS nanofibril concentration. **m** Apparent viscosity of conducting polymer inks as a function of PEDOT:PSS nanofibril concentration. **n** Shear storage modulus as a function of shear stress for conducting polymer inks of varying PEDOT:PSS nanofibril concentration. **o** Shear yield stress of conducting polymer inks as a function of PEDOT:PSS nanofibril concentration. For TEM images in (**d**–**f**), the experiments were repeated (*n* = 5) based on independently prepared samples with reproducible results. Scale bars, 100 nm.

Conducting polymers are typically used in the form of liquid monomer or polymer solution whose fluidity prevents their direct use in 3D printing^3,5,33^. In order to endow rheological properties required for 3D printing to conducting polymers, we develop a simple process to convert a commercially available PEDOT:PSS aqueous solution to a high-performance 3D printable ink (Fig. 1 and Supplementary Fig. 1). The pristine PEDOT:PSS solution exhibits a dilute dispersion of PEDOT:PSS nanofibrils (Fig. 1a, d) with low viscosity (below 30 Pa s). Inspired by 3D printability of concentrated cellulose nanofiber suspensions^34,35^, we hypothesize that a highly concentrated solution of the PEDOT:PSS nanofibrils can provide a 3D printable conducting polymer ink, due to the formation of entanglements among PEDOT:PSS nanofibrils (Fig. 1b). To test our hypothesis, we first isolate PEDOT:PSS nanofibrils by lyophilizing the pristine PEDOT:PSS solution. In order to avoid excessive formation of PEDOT-rich crystalline domains among PEDOT:PSS nanofibrils due to slow ice crystal formation during lyophilization at high temperature^36^, we perform lyophilization in a cryogenic condition (i.e., frozen in liquid nitrogen). The isolated PEDOT:PSS nanofibrils are then re-dispersed with a binary solvent mixture (water:DMSO = 85:15 v/v) to prepare concentrated suspensions.

With increasing concentration of the PEDOT:PSS nanofibrils, the suspensions gradually transit from liquids to thixotropic 3D printable inks (Fig. 1g–j) due to the formation of reversible physical networks of the PEDOT:PSS nanofibrils via entanglements within the solvent (Fig. 1e). We perform small angle X-ray scattering (SAXS) and rheological characterizations to quantify microscopic and macroscopic evolutions of the conducting polymer ink with varying concentrations of the PEDOT:PSS nanofibrils, respectively (Fig. 1k–o). The SAXS characterizations show that the average distance between PEDOT-rich crystalline domains *L* (d-spacing calculated by the Bragg expression *L* = 2π/*q*_max_) decreases with an increase in the concentration of the PEDOT:PSS nanofibrils (16.1 nm for 1 wt% and 7.0 nm for 10 wt%), indicating closer packing and higher degree of interactions between the adjacent PEDOT:PSS nanofibrils in more concentrated inks (Fig. 1k).

Rheological measurements of the conducting polymer inks clearly show the transition from low viscosity liquids (low concentration PEDOT:PSS nanofibrils) to physical gels (high concentration PEDOT:PSS nanofibrils) with characteristic shear-thinning and shear-yielding properties for 3D printable inks^18,19^ (Fig. 1l–o and Supplementary Fig. 2). The low viscosity and low yield stress of the conducting polymer inks with low PEDOT:PSS nanofibril concentrations (1-4 wt%) cause lateral spreading of 3D-printed inks on the substrate (Fig. 1g, h, m, o). On the other hand, the conducting polymer inks with too high concentrations of PEDOT:PSS nanofibrils (above 8 wt%) start to clog printing nozzles due to the formation of large aggregates of PEDOT:PSS nanofibrils (Fig. 1j, m, o). Hence, we find that the intermediate range of PEDOT:PSS nanofibril concentrations (5–7 wt%) provides optimal rheological properties and 3D printability (Fig. 1i, m, o). The 3D printable conducting polymer ink can be stored under ambient conditions over a month without the significant change in rheological properties and printability (Supplementary Fig. 3). After 3D printing, we dry and anneal the 3D-printed conducting polymers to remove solvents (water and DMSO) and facilitate the formation of PEDOT-rich crystalline domains and subsequent percolation among PEDOT:PSS nanofibrils^33^ (Fig. 1c, f) (see Methods for details). The resultant dry pure PEDOT:PSS can also be readily converted into stable pure PEDOT:PSS hydrogels (equilibrium water contents ~ 87%) by swelling in a wet environment^33^.

### 3D printing of conducting polymers

**Fig. 2:**
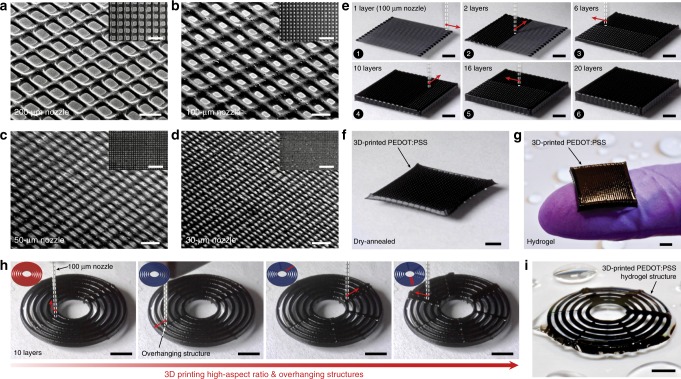
3D printing of conducting polymers. **a**–**d** SEM images of 3D-printed conducting polymer meshes by 200-µm (**a**), 100-µm (**b**), 50-µm (**c**), and 30-µm (**d**) nozzles. **e** Sequential snapshots for 3D printing of a 20-layered meshed structure by the conducting polymer ink. **f** 3D-printed conducting polymer mesh after dry-annealing. **g** 3D-printed conducting polymer mesh in hydrogel state. **h** Sequential snapshots for 3D printing of overhanging features over high aspect ratio structures by the conducting polymer ink. **i** 3D-printed conducting polymer structure with overhanging features in hydrogel state. Scale bars, 500 µm (**a**); 200 µm (**b**–**d**); 1 mm (**a**–**d**, inset panels); 2 mm (**e**–**i**).

Superior printability of the conducting polymer ink allows various advanced 3D printing capabilities including printing of high resolution, high aspect ratio, and overhanging structures (Fig. 2). To demonstrate high resolution printing in microscale, we print meshes of the conducting polymer ink (7 wt% PEDOT:PSS nanofibril) through 200-, 100-, 50-, and 30-µm diameter nozzles (Fig. 2a–d). Favorable rheological properties of the conducting polymer ink further enable the fabrication of multi-layered high aspect ratio microstructures (100-µm nozzle, 20 layers) (Supplementary Movie 1) as well as overhanging features (Supplementary Movie 2) (Fig. 2e, h). The 3D-printed conducting polymer structures can readily be converted into dry and hydrogel forms without loss of the original microscale structures, owing to the constrained drying (while attached on the substrate) and swelling property of the pure PEDOT:PSS hydrogels^33^ (Fig. 2f, g and Supplementary Fig. 4). Furthermore, the 3D-printed conducting polymer hydrogels exhibit long-term stability in physiological wet environments without observable degradation of microscale features (e.g., high aspect ratio and overhanging structures) after storing in PBS for 6 months (Supplementary Fig. 5).

The 3D printable conducting polymer ink can be readily incorporated into multi-material 3D printing processes together with other 3D printable materials. For example, we fabricate a structure that mimics a high-density multi-electrode array (MEA) based on multi-material 3D printing of the conducting polymer ink and an insulating polydimethylsiloxane (PDMS) ink with a total printing time less than 30 min (Supplementary Fig. 6a, b and Supplementary Movie 3). The 3D-printed MEA-like structure shows a complex microscale electrode pattern and a PDMS well that are comparable to a commercially available MEA fabricated by multi-step lithographic processes and post-assembly (Supplementary Fig. 6c).

### Properties of 3D-printed conducting polymers

**Fig. 3:**
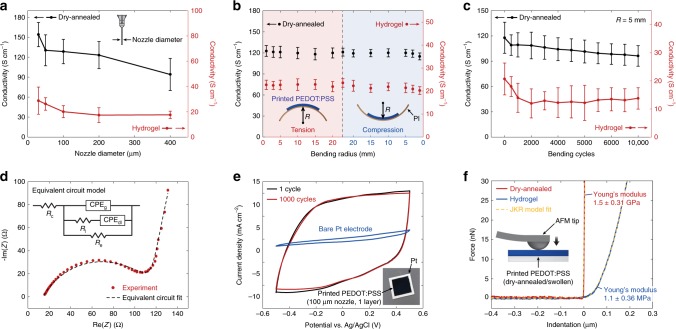
Properties of 3D-printed conducting polymers. **a** Conductivity as a function of nozzle diameter for 3D-printed conducting polymers in dry and hydrogel states. **b** Conductivity as a function of bending radius for 3D-printed conducting polymers in dry (17 µm, thickness) and hydrogel (78 µm, thickness) states. PI indicates polyimide. **c** Conductivity as a function of bending cycles for 3D-printed conducting polymers in dry (17 µm, thickness) and hydrogel (78 µm, thickness) states. **d** Nyquist plot obtained from the EIS characterization for a 3D-printed conducting polymer on Pt substrate (78 µm, thickness) overlaid with the plot predicted from the corresponding equivalent circuit model^38^. In the equivalent circuit models, *R*_e_ represents electronic resistance, *R*_i_ represents ionic resistance, *R*_c_ represents the total ohmic resistance of the cell assembly, CPE_dl_ represents the double-layer constant phase element (CPE), whereas CPE_g_ represents the geometric CPE. CPE is used to account inhomogeneous or imperfect capacitance and are represented by the parameters *Q* and *n* where *Q* represents the peudocapacitance value and *n* represents the deviation from ideal capacitive behavior. The true capacitance *C* can be calculated from these parameters by using the relationship *C* = *Q*ω_max_^*n*−1^, where ω_max_ is the frequency at which the imaginary component reaches a maximum^37^. The fitted values for 3D-printed PEDOT:PSS are *R*_e_ = 107.1 Ω, *R*_i_ = 105.5 Ω, *R*_c_ = 14.07 Ω, *Q*_dl_ = 1.467 × 10^−5^ F s^*n*−1^, *n*_dl_ = 0.924, *Q*_g_ = 4.446 × 10^−7^ F s^*n*−1^, and *n*_dl_ = 0.647. **e** CV characterization for a 3D-printed conducting polymer on Pt substrate. **f** Nanoindentation characterizations for 3D-printed conducting polymers in dry and hydrogel states with the JKR model fits. Values in (**a**–**c**) represent the mean and the standard deviation (*n* = 5 per each testing conditions based on independently prepared samples and performed experiments).

The 3D-printed conducting polymers can achieve electrical conductivity as high as 155 S cm^−1^ in the dry state and 28 S cm^−1^ in the hydrogel state, comparable to the previously reported high-performance conducting polymers^5,16,33^ (Fig. 3a, Supplementary Fig. 7, and Supplementary Table 2). Notably, a smaller nozzle diameter yields a higher electrical conductivity for the printed conducting polymers, potentially due to shear-induced enhancements in the PEDOT:PSS nanofibril alignment^22^. Flexibility of the 3D-printed conducting polymers allows mechanical bending with maximum strain of 13% in the dry state (65 µm radius of curvature with 17 µm thickness) and 20% in the hydrogel state (200 µm radius of curvature with 78 µm thickness) without failure (Supplementary Fig. 8). To investigate the effect of mechanical bending on the electrical performance, we characterize the electrical conductivity of the 3D-printed conducting polymers (100-µm nozzle, 1 layer) on flexible polyimide substrates as a function of the bending radius as well as the bending cycle (Fig. 3b, c). The 3D-printed conducting polymers show small changes in the electrical conductivity (less than 5%) across a wide range of tensile and compressive bending conditions (radius of curvature, ±1–20 mm) in both the dry and hydrogel states (Fig. 3b). Furthermore, the 3D-printed conducting polymers can maintain a high electrical conductivity (over 100 S cm^−1^ in dry state and over 15 S cm^−1^ in hydrogel state) after 10,000 cycles of repeated bending (Fig. 3c).

To further investigate the electrical properties, we perform the electrochemical impedance spectroscopy (EIS) of the 3D-printed conducting polymers (100-µm nozzle, 1 layer on Pt) (Fig. 3d). The EIS data are fitted to the equivalent circuit model shown in Fig. 3d, where *R*_e_ represents the electronic resistance, *R*_i_ represents the ionic resistance, *R*_c_ represents the total ohmic resistance of the electrochemical cell assembly, and CPE_dl_ and CPE_g_ represent the constant phase elements (CPE) corresponding to the double-layer ionic capacitance and the geometric capacitance, respectively^37,38^. The semicircular Nyquist plot shape suggests the presence of comparable ionic and electronic conductivity in the 3D-printed conducting polymer hydrogels (Fig. 3d), which is confirmed by the extracted fitting parameters of the equivalent circuit model where the ionic and electronic resistances show comparable magnitudes (*R*_i_ = 105.5 Ω and *R*_e_ = 107.1 Ω).

The cyclic voltammetry (CV) demonstrates a high charge storage capability (CSC) of the 3D-printed conducting polymers (100-µm nozzle, 1 layer on Pt) compared to typical metallic electrode materials such as Pt with remarkable electrochemical stability (less than 2% reduction in CSC after 1000 cycles) (Fig. 3e). The CV of the 3D-printed conducting polymers further shows broad and stable anodic and cathodic peaks under varying potential scan rates^39^, suggesting non-diffusional redox processes and electrochemical stability of the 3D-printed conducting polymers (Supplementary Fig. 9).

To quantify mechanical properties of the 3D-printed conducting polymers, we conduct nanoindentation tests (Fig. 3f and Supplementary Fig. 10). The 3D-printed conducting polymers display relatively high Young’s modulus of 1.5 ± 0.31 GPa in the dry state, similar to the previously reported values for dry PEDOT:PSS^40^ (Fig. 3f). In contrast, the 3D-printed conducting polymers in the hydrogel state exhibit three orders of magnitude reduction in Young’s modulus to 1.1 ± 0.36 MPa, comparable to those of soft elastomers such as PDMS (Young’s modulus, 1–10 MPa) (Fig. 3f). The softness of 3D-printed conducting polymer hydrogels can offer favorable long-term biomechanical interactions with biological tissues, which may find a particular advantage in bioelectronic devices and implants^3,41,42^.

### 3D printing of conducting polymer devices

**Fig. 4:**
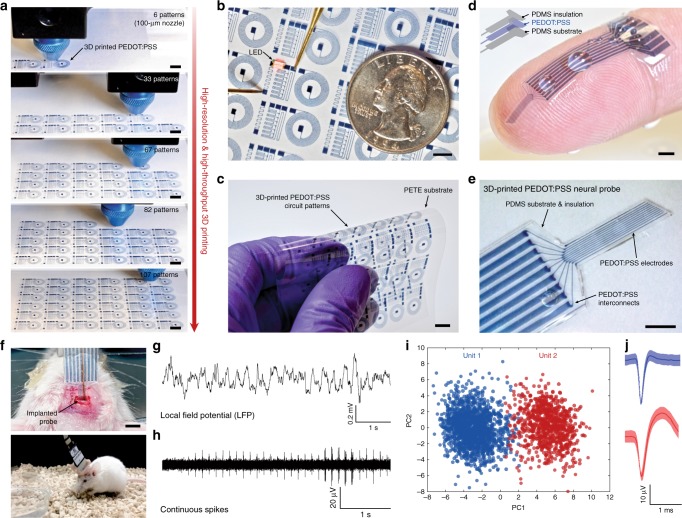
3D printing of conducting polymer devices. **a** Sequential snapshots for 3D printing of high-density flexible electronic circuit patterns by the conducting polymer ink. **b** Lighting up of LED on the 3D-printed conducting polymer circuit. PETE indicates polyethylene terephthalate. **c** Bending of the 3D-printed conducting polymer circuit without failure. **d** Image of the 3D-printed soft neural probe with 9-channels by the conducting polymer ink and the PDMS ink. **e** Image of the 3D-printed soft neural probe in magnified view. **f** Images of the implanted 3D-printed soft neural probe (top) and a freely moving mouse with the implanted probe (bottom). **g**, **h** Representative electrophysiological recordings in the mouse dHPC by the 3D-printed soft neural probe. Local field potential (LFP) traces (0.5 to 250 Hz) under freely moving conditions (**g**). Continuous extracellular action potential (AP) traces (300 to 40 kHz) recorded under freely moving conditions (**h**). **i** Principal component analysis of the recorded single-unit potentials from (**h**). **j** Average two units spike waveforms recorded over time corresponding to clusters in (**i**). Scale bars, 5 mm (**a**–**c**); 1 mm (**d**, **e**); 2 mm (**f**).

Enabled by the superior 3D printability and properties, 3D printing of the conducting polymer ink can offer a promising route for facile and streamlined fabrication of high resolution and multi-material conducting polymer structures and devices (Fig. 4 and Supplementary Table 1). Highly reproducible 3D printing of conducting polymers in high resolution allows the rapid fabrication of over 100 circuit patterns with less than 100 µm feature size on a flexible polyethylene terephthalate (PETE) substrate by a single continuous printing process with a total printing time less than 30 min (Fig. 4a, Supplementary Fig. 11, and Supplementary Movie 4). The resultant 3D-printed conducting polymer electronic circuits exhibit high electrical conductivity to operate electrical components such as a light emitting diode (LED) (Fig. 4b and Supplementary Movie 4) and flexibility to withstand bending without mechanical failure (Fig. 4c). This programmable, high resolution, and high throughput fabrication of conducting polymer patterns by 3D printing can potentially serve as an alternative to ink-jet printing and screen printing with a higher degree of flexibility in the choice of designs based on applicational demands^4,7^.

We further demonstrate a facile fabrication of a soft neural probe for in vivo bioelectronic signal recording (Supplementary Fig. 12 and Supplementary Movie 5). The multi-material 3D printing capability in high resolution allows us to print both insulating encapsulation (PDMS ink) and electrodes (conducting polymer ink) of the neural probe by a facile continuous printing process (a total printing time less than 20 min) without the need of post-assemblies or complex multi-step procedures in conventional fabrication methods such as electron-beam lithography^15,16^ (Fig. 4d, Supplementary Fig. 12, and Supplementary Movie 5). The resultant probe consists of nine PEDOT:PSS electrode channels in the feature size of 30 µm in diameter with the impedance in the range of 50–150 kΩ at 1 kHz, suitable for in vivo recording of neural activities^43,44^. After the connector assembly (Supplementary Fig. 13), the 3D-printed soft neural probe is implanted to the mouse dorsal hippocampus (dHPC, coordinate: −1.8 mm AP; 1.5 mm ML; −1.0 mm DV) with the help of a plastic catheter (Fig. 4f, top). The 3D-printed soft neural probe can successfully record continuous neural activities in a freely moving mouse (Fig. 4f, bottom) from each channel including the local field potential (LFP; at 1 kHz) (Fig. 4g) and the action potential (AP; at 40 kHz) (Fig. 4h) over two weeks. Furthermore, the 3D-printed soft neural probe can record signals from distinctive single units, isolated from individual channel of the probe (Fig. 4i, j).

## Discussion

In summary, we present a high-performance 3D printable conducting polymer ink based on PEDOT:PSS capable of rapid and flexible fabrication of highly conductive microscale structures and devices both in the dry and hydrogel states. The conducting polymer ink exhibits superior 3D printability and ready integrability into advanced multi-material 3D printing processes with other 3D printable materials. Enabled by this capability, we further demonstrate 3D printing-based fabrication of the high-density flexible electronic circuit and the soft neural probe in a facile, fast, and significantly streamlined manner. This work not only addresses the existing challenges in 3D printing of conducting polymers but also offers a promising fabrication strategy for flexible electronics, wearable devices, and bioelectronics based on conducting polymers.

## Methods

### Preparation of 3D printable conducting polymer ink

A commercially available PEDOT:PSS aqueous solution (Clevios^TM^ PH1000, Heraeus Electronic Materials) was stirred vigorously for 6 h at room temperature and filtered with a syringe filter (0.45 µm). The filtered pristine PEDOT:PSS solution was then cryogenically frozen by submerging in liquid nitrogen bath. The cryogenically frozen PEDOT:PSS solution was lyophilized for 72 h to isolate PEDOT:PSS nanofibrils. The isolated PEDOT:PSS nanofibrils with varying concentrations were re-dispersed with a deionized water-DMSO (Sigma-Aldrich) mixture (water:DMSO = 85:15 v/v), followed by thorough mixing and homogenization by a mortar grinder (RM 200, Retcsh). The prepared conducting polymer ink was kept at 4 °C before use. The detailed procedure for the 3D printable conducting polymer ink preparation is illustrated in Supplementary Fig. 1.

### 3D printing procedure

3D printing of the conducting polymer ink and the PDMS ink (SE 1700, Dow Corning) were conducted based on a custom-designed 3D printer based on a Cartesian gantry system (AGS1000, Aerotech)^18^ with various size of nozzles (200- and 100-µm nozzles from Nordson EFD; 50-µm nozzles from Fisnar; 30-µm nozzles from World Precision Instrument). Printing paths were generated by CAD drawings (SolidWorks, Dassault Systèmes) and converted into G-code by a commercial software package (CADFusion, Aerotech) and custom Python scripts to command the x-y-z motion of the printer head. The detailed printing paths are provided in Supplementary Figs. 6, 11, and 12.

After printing, the 3D-printed conducting polymer was dried at 60 °C for 24 h followed by multiple cycles of annealing at 130 °C (3 cycles with 30 min per each cycle) to yield pure PEDOT:PSS^33^. To achieve constrained drying of 3D-printed conducting polymers in thickness direction, the 3D-printed conducting polymers were placed on a glass substrate and dry-annealed. The dry-annealed 3D-printed conducting polymer was further equilibrated in PBS to be converted into a hydrogel state.

### Electron microscope imaging

Scanning electron microscope (SEM) images of the 3D-printed conducting polymers were taken by using a SEM facility (JSM-6010LA, JEOL) with 5 nm gold sputtering to enhance image contrasts. Transmission electron microscope (TEM) images of the pristine PEDOT:PSS solution, conducting polymer ink, and dry-annealed 3D-printed conducting polymer were taken by using a TEM facility (2100 FEG, JEOL) at 200 kV with a magnification of 10,000 to 60,000. For cryogenic TEM (CryoTEM) imaging, the samples were prepared by a cryo plunger (CP3, Gatan). All Images were recorded under low-dose conditions to avoid sample damage by electron beams.

Small angle X-ray scattering (SAXS) characterizations of the conducting polymer inks were conducted by using a SAXS facility (Pilatus 3 R 300 K, Bruker Nanostar SAXS) with a sample-detector distance of 1059.1 mm and an exposure time of 300 s. The measured scattering intensity was corrected by subtracting the solvent background (water:DMSO = 85:15 v/v). To analyze the average distance between PEDOT crystalline domains in the 3D printable conducting polymer inks, the d-spacing *L* was calculated by the Bragg expression *L* = 2π/*q*_max_ without further fitting of the SAXS data.

### Rheological characterization

Rheological characterizations of the conducting polymer inks were conducted by using a rotational rheometer (AR-G2, TA Instrument) with 20-mm diameter steel parallel-plate geometry. Apparent viscosity was measured as a function shear rate by steady-state flow tests with a logarithmic sweep of shear rate (0.01–100 s^−1^). Shear storage modulus (*G*′) and loss modulus (*G*″) were measured as a function of shear stress via oscillation tests with a logarithmic sweep of shear stress (1–1000 Pa) at 1 Hz shear frequency and oscillatory strain of 0.02. Shear yield stress for each sample was identified as a shear stress at which shear and loss moduli were the same values. All rheological characterizations were conducted at 25 °C with preliminary equilibration time of 1 min.

### Nanoindentation

Nanoindentation characterizations of 3D-printed conducting polymers were conducted by using an atomic force microscope (AFM) facility (MFP-3D, Asylum Research) with indentation depth of 50 nm (for dry state) and 1 µm (for hydrogel state). A spherical tip with 50 nm radius (biosphere^TM^, Asylum Research) was used for the nanoindentation measurements. Young’s moduli of the samples were obtained by fitting force vs. indentation curve with a JKR model^45^ (Fig. 3f).

### Electrical conductivity measurement

Electrical conductivity of the 3D-printed conducting polymers was measured by using a standard four-point probe (Keithley 2700 digital multimeter, Keithley). To prepare conductivity measurement samples, one layer of the conducting polymer ink was printed into a rectangular shape (30 mm in length and 5 mm in width) with 100-µm nozzles on glass substrates (17 µm and 78 µm in thickness for dry-annealed and hydrogel samples, respectively). Copper wire electrodes (diameter, 0.5 mm) were attached onto the surface of dry-annealed 3D-printed conducting polymer by applying silver paste, while platinum wire electrodes (diameter, 0.5 mm) were employed for hydrogels to avoid the corrosion in wet environments (Supplementary Fig. 7). The electrical conductivity *σ* of the samples was calculated as

$${\upsigma} = \frac{{I \times L}}{{V \times W \times T}},$$

where *I* is the current flowing through the sample, *L* is the distance between the two electrodes for voltage measurement, *V* is the voltage across the electrodes, *W* is the width of the sample, and *T* is the thickness of the sample.

For electrical conductivity measurements under cyclic bending, one layer of the conducting polymer ink was printed into a rectangular shape (30 mm in length and 5 mm in width) with 100-µm nozzles on polyimide substrates (17 µm and 78 µm in thickness for dry-annealed and hydrogel samples, respectively). Cyclic bending of the sample was performed by using a custom-made fixture with controllable bending radius of curvature.

### Electrochemical measurement

Cyclic voltammetry (CV) of the 3D-printed conducting polymer was performed by using a potentiostat/galvanostat (VersaSTAT 3, Princeton Applied Research) with a range of scan rates (50 to 500 mV s^−1^). Pt wires (diameter, 1 mm) were employed as both working and counter electrodes, and an Ag/AgCl electrode was used as the reference electrode. Prior to all measurements, the working and counter electrodes were cleaned successively with abrasive paper, deionized water, and ethyl alcohol. PBS was used as the supporting electrolyte.

Electrochemical impedance spectroscopy (EIS) measurements of the 3D-printed conducting polymer were carried out by using a potentiostat/galvanostat (1287 A, Solartron Analytical) and a frequency response analyzer (1260 A, Solatron Analytical) in an electrochemical cell installed with Pt sheet as both working and counter electrodes and Ag/AgCl as a reference electrode. The frequency range between 0.1 and 100 kHz was scanned in PBS with an applied bias of 0.01 V vs. Ag/AgCl. The EIS data for the 3D-printed conducting polymer were fitted by using an equivalent circuit model for further analysis (Fig. 3d).

### In vivo electrophysiology by 3D-printed soft neural probe

Young adult mice (60-70 days old, Balb/C Male Jackson Laboratory Stock # 000651) were used in the electrophysiological experiments. Mice were maintained under a 12 h light/dark cycle at 22–25 °C, and given ad libitum access to tap water and standard chow. All procedures were approved by the Animal Advisory Committee at Zhejiang University and followed the US National Institutes of Health Guidelines for the Care and Use of Laboratory Animals. For all surgeries, mice were anesthetized with 1% pentobarbital (Sigma-Aldrich), and then fixed in a stereotaxic frame. A craniotomy was performed at −1.80 mm anterior to bregma and 1.5 mm lateral to the midline. The incision was closed with tissue glue (VetBond^TM^, 3 M). Electrophysiological recording in the dHPC was carried out by using the 3D-printed soft neural probe coupling with Neuro Nano Strip Connectors (Omnetics). All data shown in Fig. 4g–j were collected from BALB/c mice in the dHPC with a 64-channel multi-electrode recording system (Plexon). After the probe implantation, mice were allowed to recover for at least 3 days. Neuronal signals were referenced to two connected skull screws (above the prefrontal cortex and cerebellum). Spike sorting was carried out in Offline Sorter software (Plexon). In the principal component analysis, a rough separation of units from PNs and interneurons in the dHPC was mainly based on their differences in spike wave shapes and mean baseline firing rates.

### Reporting summary

Further information on research design is available in the Nature Research Reporting Summary linked to this article.

## Supplementary information

Supplementary Information

Description of Additional Supplementary Files

Supplementary Movie 1

Supplementary Movie 2

Supplementary Movie 3

Supplementary Movie 4

Supplementary Movie 5

Reporting Summary

## Data Availability

The data that support the findings of this study are available from the corresponding author upon reasonable request.

## Code availability

The custom codes used this study for 3D printing are available from the corresponding author upon reasonable request.
