# Supplementary material for: 3D printing of conducting polymers
Source: Nat Commun. 2020 Mar 30;11:1604. doi: 10.1038/s41467-020-15316-7 (PMC7105462; doi:10.1038/s41467-020-15316-7)
Supplement: Supplementary file 1 — Supplementary Information [file 41467_2020_15316_MOESM1_ESM.pdf]

3D Printing of Conducting Polymers

Yuk et al.

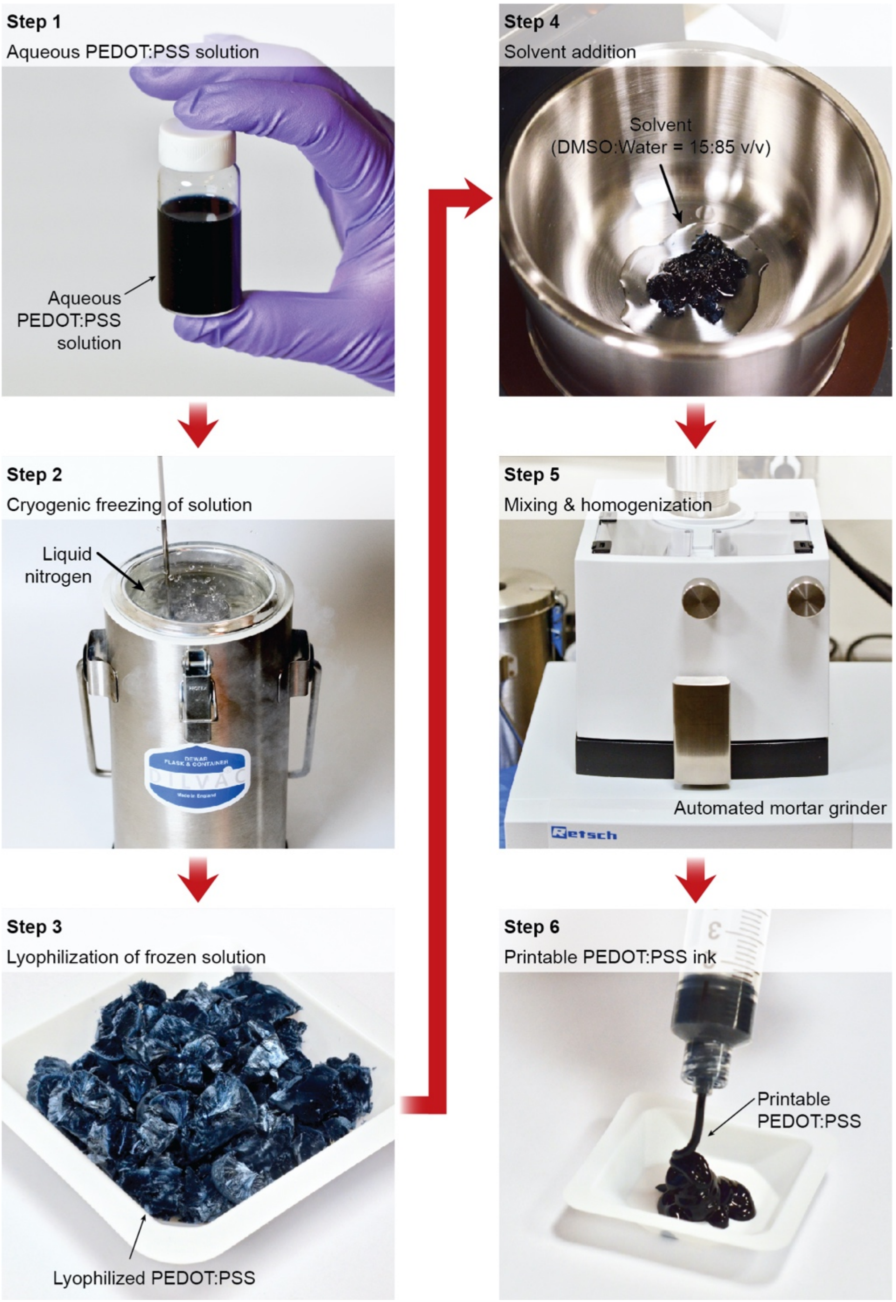

Supplementary Figure 1 | Preparation of 3D printable conducting polymer ink. Step 1, Stirring and filtration of a pristine PEDOT:PSS aqueous solution; Step 2, Cryogenic freezing of the PEDOT:PSS solution in a liquid nitrogen bath; Step 3, Lyophilization of the cryogenically frozen PEDOT:PSS solution to isolate PEDOT:PSS nanofibrils; Step 4, Re-dispersion of the PEDOT:PSS nanofibrils with a solvent mixture (water:DMSO = 85:15 v/v); Step 5, Mixing and homogenization by using a mortar grinder; Step 6, The resultant homogeneous 3D printable conducting polymer ink.

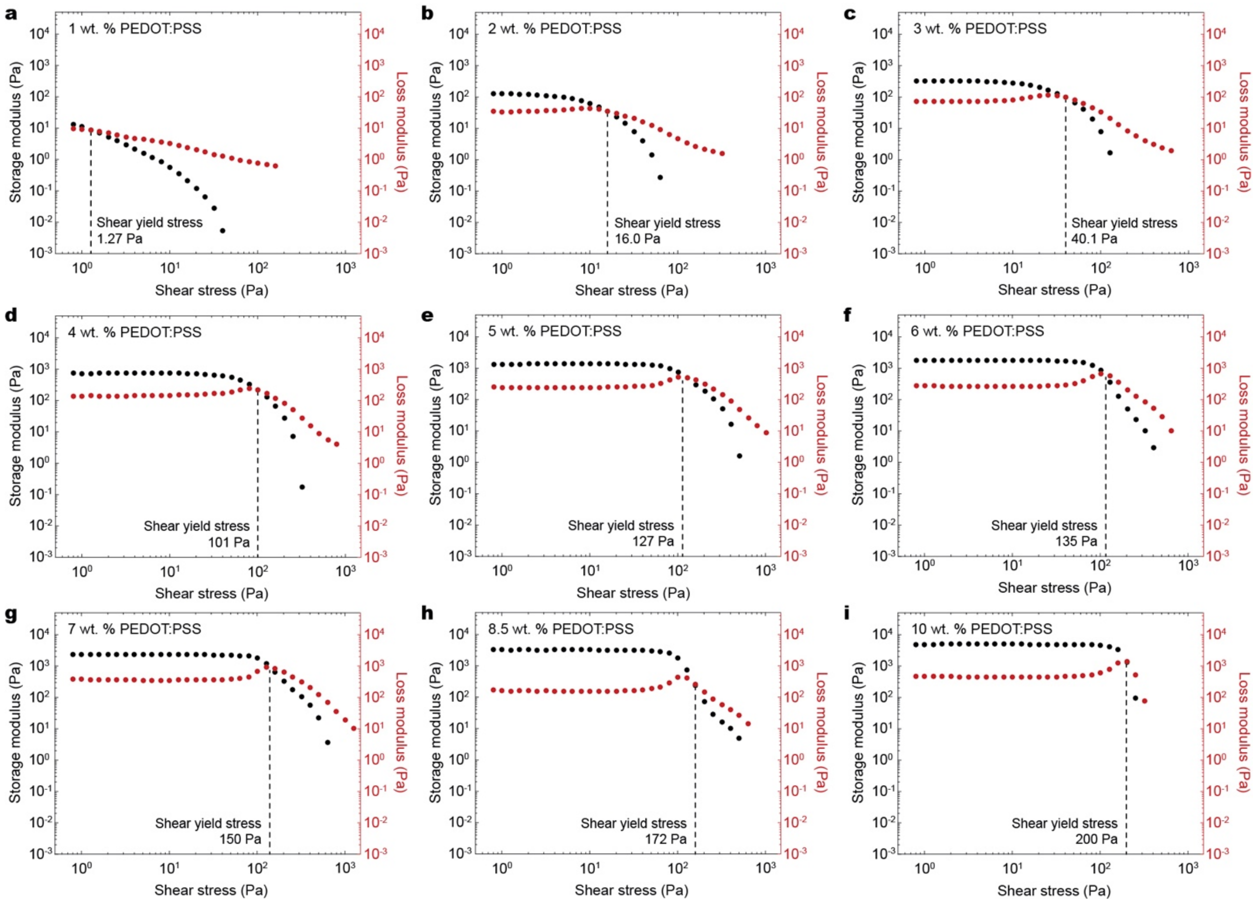

Supplementary Figure 2 | Rheological characterizations of conducting polymer inks with varying PEDOT:PSS nanofibril concentration. a-i, Storage and loss moduli as a function of shear stress for conducting polymer inks with PEDOT:PSS nanofibril concentration of 1 wt. % (a), 2 wt. % (b), 3 wt. % (c), 4 wt. % (d), 5 wt. % (e), 6 wt. % (f), 7 wt. % (g), 8.5 wt. % (h), and 10 wt. % (i). Shear yield stress for each ink was identified as a shear stress at which shear and loss moduli were the same values.

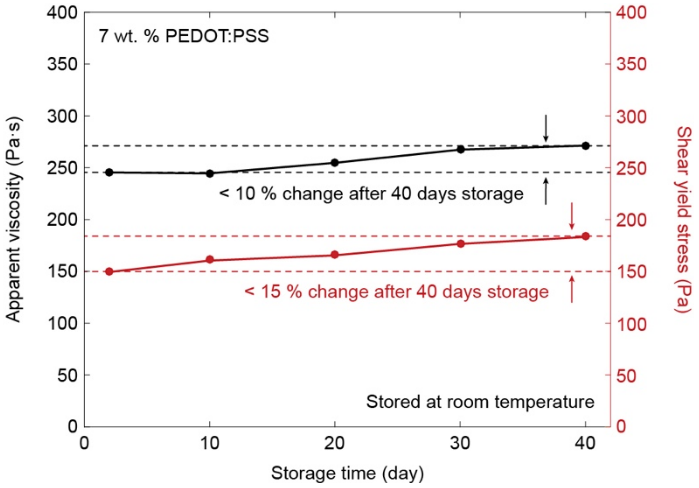

Supplementary Figure 3 | Rheological stability of conducting polymer ink. Apparent viscosity (black) and shear yield stress (red) of the conducting polymer ink with 7 wt. % PEDOT:PSS nanofibril concentration showed good stability over 40 days of storage at room temperature.

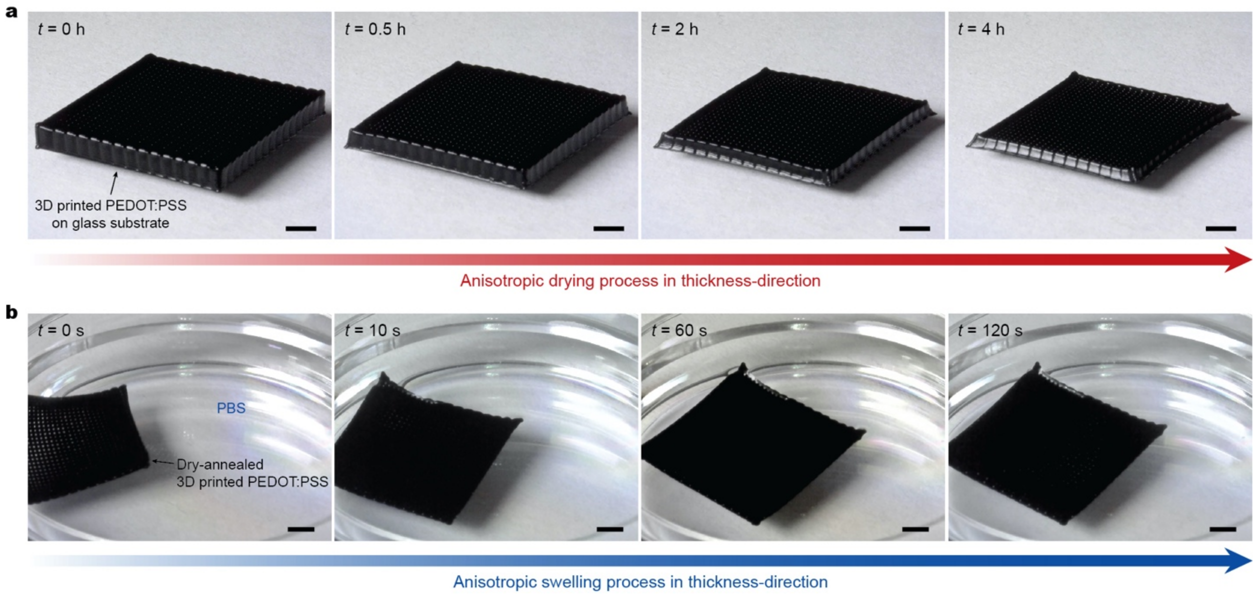

Supplementary Figure 4 | Constrained drying and swelling processes of 3D-printed conducting polymer structure. a, Constrained drying in thickness direction of a 20-layered 3D-printed conducting polymer mesh on a glass substrate in ambient condition. **b**, Swelling in thickness direction of the dried 3D-printed conducting polymer mesh in PBS. Scale bars, 2 mm

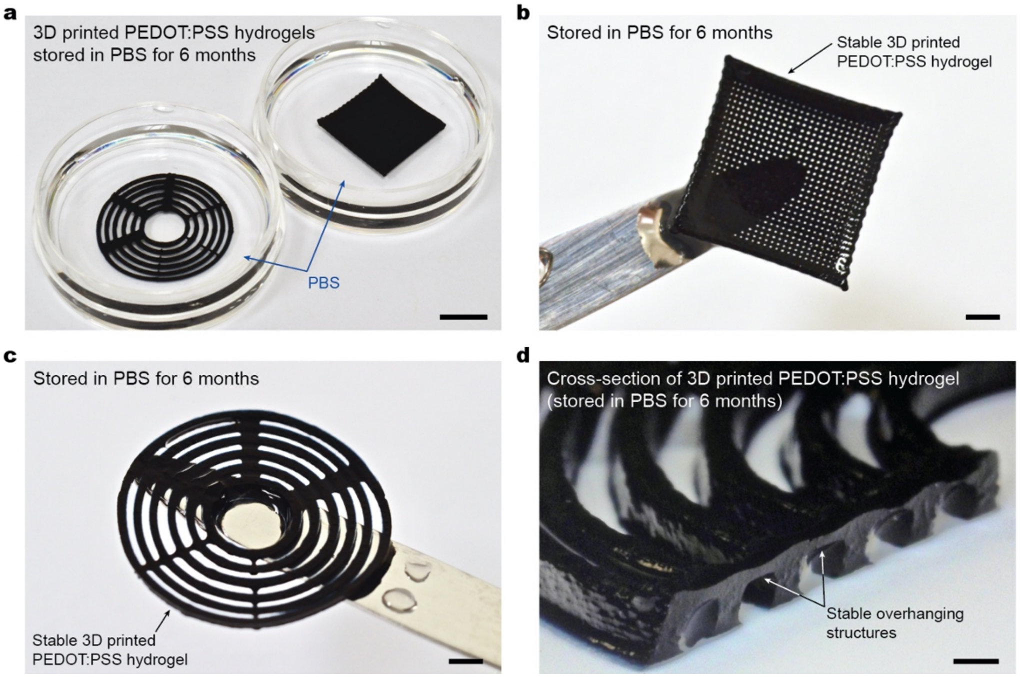

Supplementary Figure 5 | Long-term stability of 3D-printed conducting polymer hydrogels. **a**, Stable 3D-printed conducting polymer hydrogels stored in PBS for 6 months. **b**,**c**, Close up view of the 3D-printed mesh (b) and the overhanging (c) hydrogel structures stored in PBS for 6 months. **d**, Cross-section of the 3D-printed conducting polymer hydrogel stored in PBS for 6 months with stable overhanging structures. Scale bars, 5 mm (**a**); 2 mm (**b**, **c**); 500 µm (**d**)

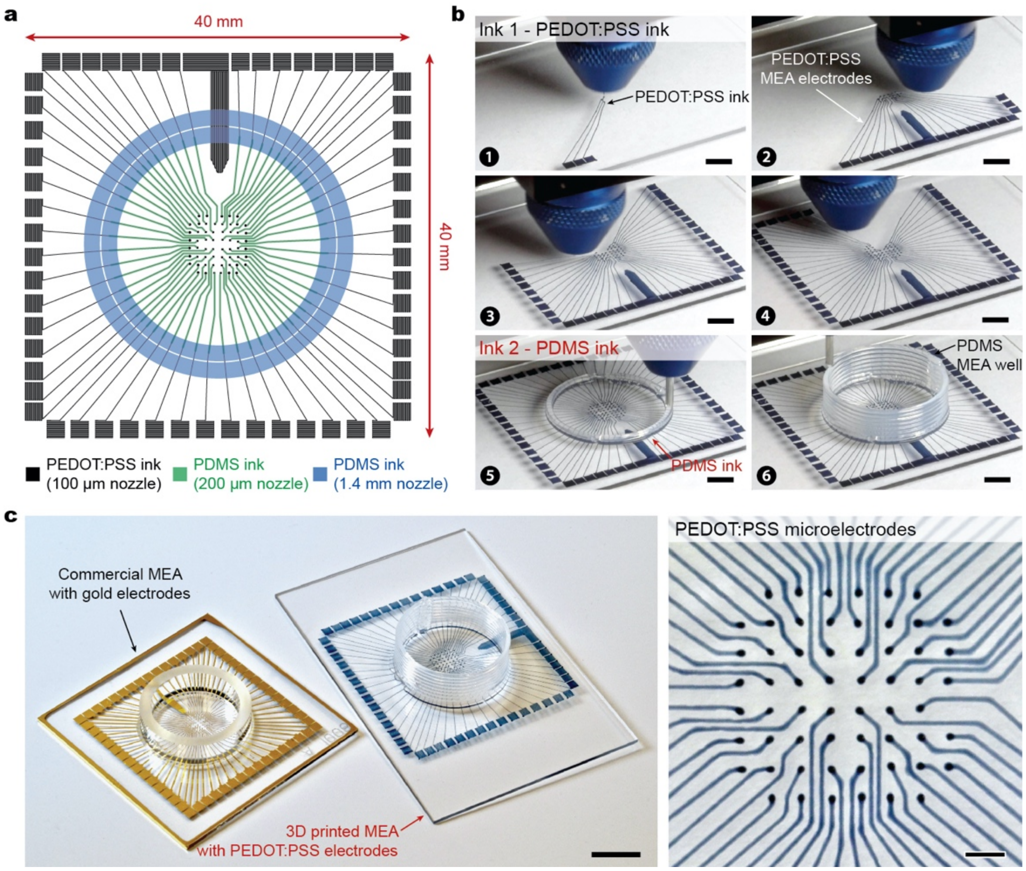

Supplementary Figure 6 | Multi-material 3D printing of MEA. a, Design and printing paths for a MEA with 60 electrodes and a culture well. **b**, Sequential snapshots for 3D printing of the MEA based on the conducting polymer ink and the PDMS ink. **c**, Image of the 3D-printed MEA placed next to a commercially-available MEA with the same design fabricated by multi-step lithographic processes and post assembly (left). Magnified view of the 3D-printed conducting polymer microelectrodes (right). Scale bars, 5 mm (**b**); 10 mm (**c**, left panel); 1 mm (**c**, right panel)

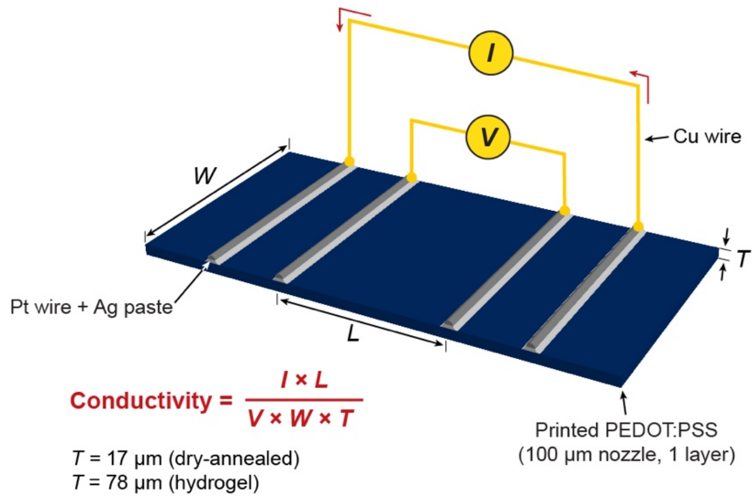

Supplementary Figure 7 | Measurement setup for electrical conductivity of 3D-printed conducting polymers. Four-point probe setup for electrical conductivity measurement of 3D-printed conducting polymer in dry-annealed or hydrogel states.

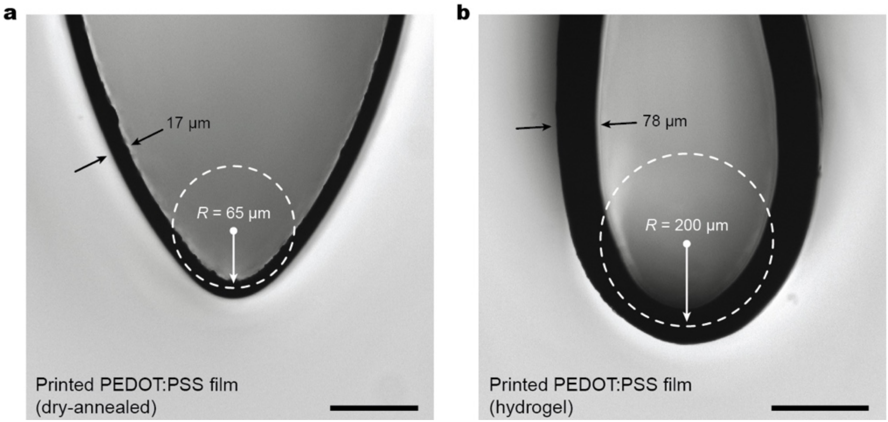

Supplementary Figure 8 | Flexibility of 3D-printed conducting polymers. a, Bending of a 3D-printed conducting polymer (thickness, 17 µm) in dry state with radius of curvature of 65 µm. **b**, Bending of a 3D-printed conducting polymer (thickness, 78 µm) in hydrogel state with radius of curvature of 200 µm. Experiments were repeated (n = 5) based on independently prepared samples with reproducible results. Scale bars, 100 µm (**a**); 200 µm (**b**)

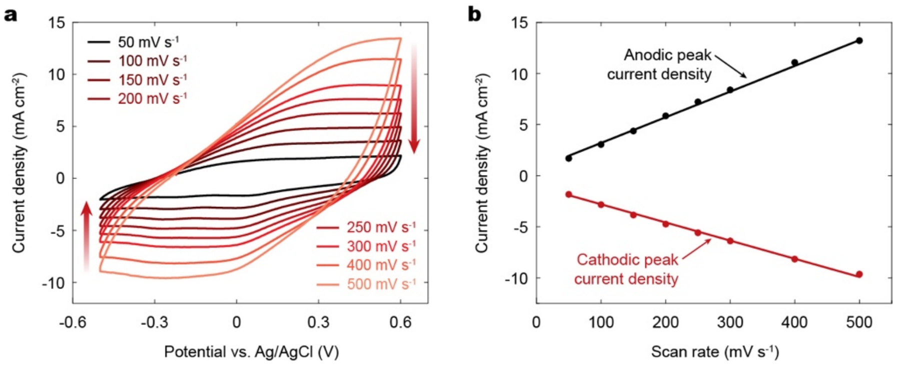

Supplementary Figure 9 | CV characterization of 3D-printed conducting polymers at varying scan rate. a, CV characterizations of the 3D-printed conducting polymer on Pt substrate at varying potential scan rates from 500 to 50 mV s^{-1}. **b**, Anodic and cathodic peak current densities as a function of potential scan rates during the CV characterizations.

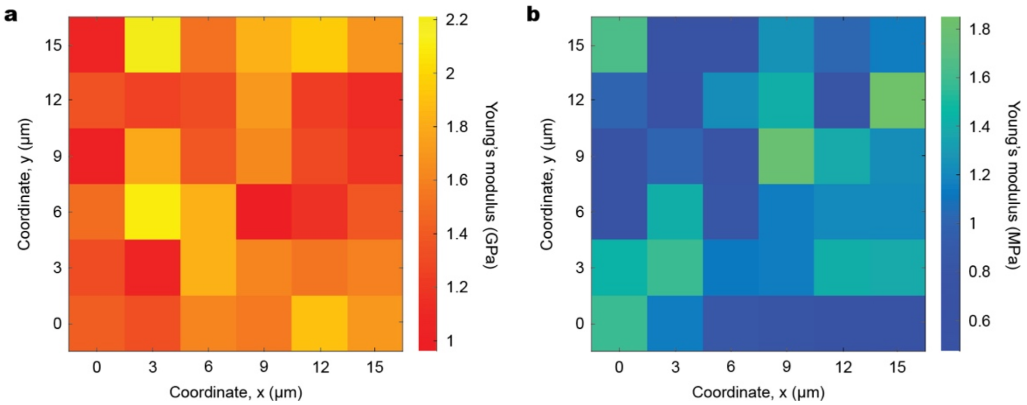

Supplementary Figure 10 | Young's moduli map for 3D-printed conducting polymers. a,**b**, Young's moduli map of a 3D-printed conducting polymer in dry (a) and hydrogel (b) states measured by nanoindentation.

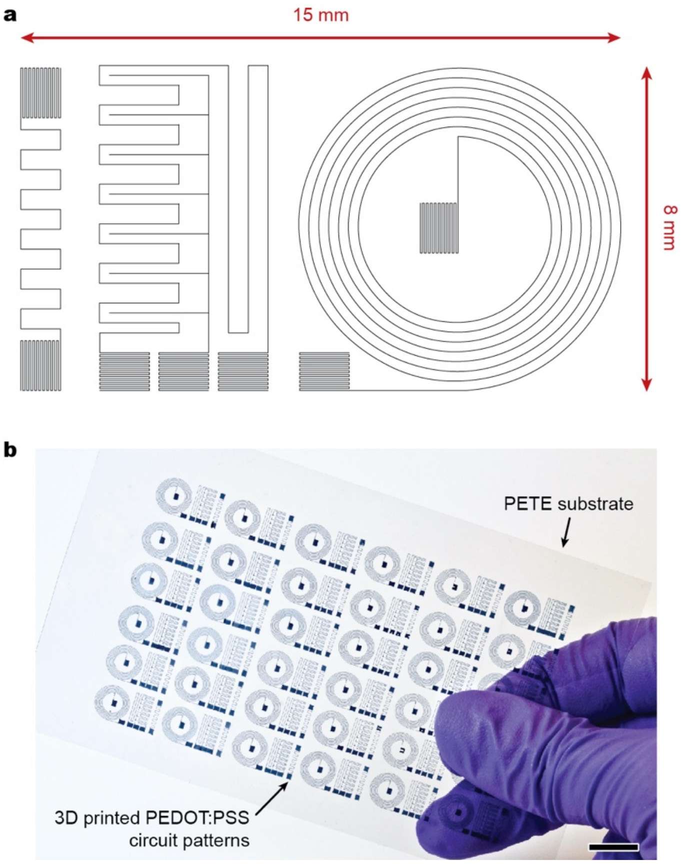

Supplementary Figure 11 | High resolution, high throughput 3D printing of flexible circuit patterns. **a**, Design and printing paths for circuits. **b**, Image of 3D-printed conducting polymer circuit patterns (108 patterns) on a flexible PETE substrate. Scale bar, 10 mm

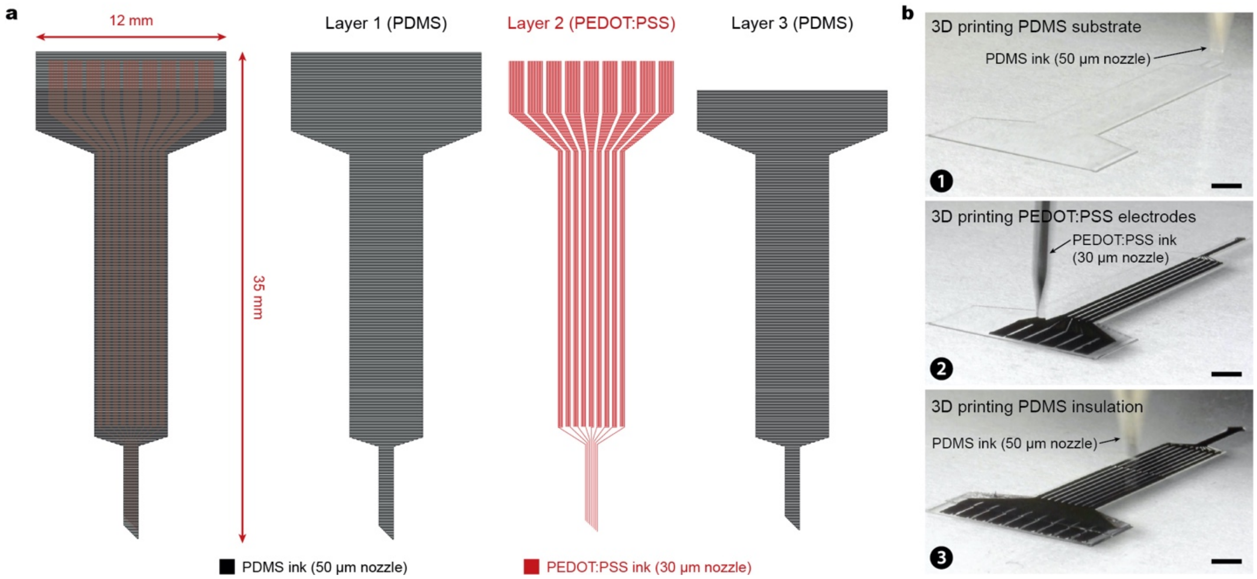

Supplementary Figure 12 | Multi-material 3D printing of soft neural probe. a, Design and printing paths for a soft neural probe with 9 electrode channels and insulating layers. **b**, Sequential snapshots for 3D printing of the soft neural probe based on the conducting polymer ink and the PDMS ink. Scale bars, 2 mm

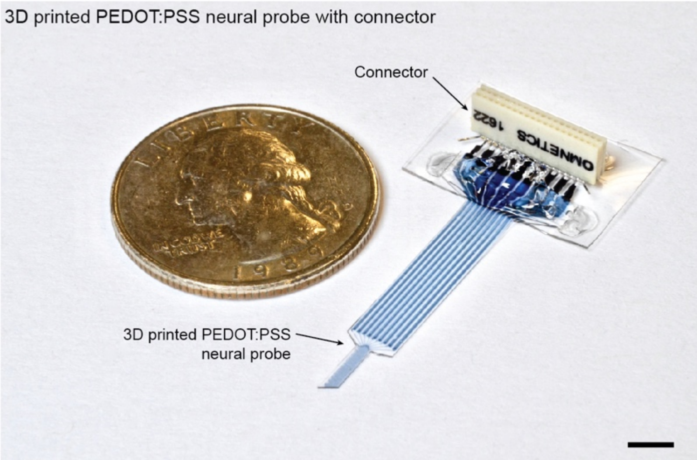

Supplementary Figure 13 | Connector-assembled 3D-printed soft neural probe. A multi-channel connector assembled with the 3D-printed soft neural probe for communication with electrophysiology measurement systems. Scale bar, 2 mm

Supplementary Table 1 | Comparison of various fabrication methods for conducting polymers

Printing method	Resolution	Structure dimension	Multi-material compatibility	Fabrication complexity	References
Aerosol printing	> 10 μm	2D	Yes (low viscosity solutions)	Moderate (carrier-sheath gas flow systems)	*1-3*
Ink-jet printing	> 50 μm	2D	Yes (low viscosity solutions)	Low	*4-9*
Screen printing	> 200 μm	2D	Yes (high viscosity solutions)	Low	*10-12*
Lithography	> 10 μm	2D	Yes (lithography-compatible materials)	High (masking & etching processes)	*13-16*
Electrochemical patterning	> 100 μm	2D	No	Low	*17-19*
**This work**	**> 30 μm**	**2D or 3D**	**Yes (3D printable inks)**	**Low**	

Supplementary Table 2 | Comparison of electrical conductivity for various PEDOT:PSS materials

Material	State	Conductivity	Preparation method	Reference
PEDOT:PSS + DMSO[a]	Dry	1,500 S cm^{-1}	Spin-coating & post-treatment with DMSO	*20*
PEDOT:PSS + EG[b]	Dry	1,330 S cm^{-1}	Spin-coating & post-treatment with EG	*21*
PEDOT:PSS + MSA[c]	Dry	3,300 S cm^{-1}	Spin-coating & post-treatment with MSA	*22*
PEDOT:PSS + ionic liquid	Dry	3,100 S cm^{-1}	Spin-coating & post-treatment with ionic liquid	*7*
PEDOT:PSS + H_2SO_4	Hydrogel	8.8 S cm^{-1}	Molding & post-treatment with H_2SO_4	*23*
PEDOT:PSS + ionic liquid + PAAc[d]	Hydrogel	0.23 S cm^{-1}	Molding & removal of ionic liquid in water	*24*
PEDOT:PSS + ionic liquid	Hydrogel	47 S cm^{-1}	Spin-coating or lithography & removal of ionic liquid in water	*16*
PEDOT:PSS + Cu	Hydrogel	0.23 S cm^{-1}	Electrogelation & removal of Cu in water	*19*
PEDOT:PSS + DMSO	Hydrogel	40 S cm^{-1}	Casting & removal of water and DMSO by dry-annealing	*25*
**This work**	**Dry & Hydrogel**	**155 S cm^{-1} (dry)<br>28 S cm^{-1} (hydrogel)**	**3D printing & removal of water and DMSO by dry-annealing**	

[a]DMSO: Dimethyl sulfoxide
[b]EG: Ethylene glycol
[c]MSA: Methanesulfonic acid
[d]PAAc: Poly(acrylic acid)

Supplementary References

1 Hong, K., Kim, S. H., Mahajan, A. & Frisbie, C. D. Aerosol jet printed p-and n-type electrolyte-gated transistors with a variety of electrode materials: Exploring practical routes to printed electronics. *ACS Applied Materials & Interfaces* **6**, 18704-18711 (2014).

2 Hong, K. *et al.* Aerosol Jet Printed, Sub-2 V Complementary Circuits Constructed from P- and N-Type Electrolyte Gated Transistors. *Advanced Materials* **26**, 7032-7037 (2014).

3 Thompson, B. & Yoon, H.-S. Aerosol-printed strain sensor using PEDOT: PSS. *IEEE Sensors Journal* **13**, 4256-4263 (2013).

4 Sirringhaus, H. *et al.* High-resolution inkjet printing of all-polymer transistor circuits. *Science* **290**, 2123-2126 (2000).

5 Wang, J., Zheng, Z., Li, H., Huck, W. & Sirringhaus, H. Dewetting of conducting polymer inkjet droplets on patterned surfaces. *Nature Materials* **3**, 171 (2004).

6 Pan, L. *et al.* Hierarchical nanostructured conducting polymer hydrogel with high electrochemical activity. *Proceedings of the National Academy of Sciences* **109**, 9287-9292 (2012).

7 Wang, Y. *et al.* A highly stretchable, transparent, and conductive polymer. *Science Advances* **3**, e1602076 (2017).

8 Bihar, E. *et al.* Inkjet-printed PEDOT: PSS electrodes on paper for electrocardiography. *Advanced Healthcare Materials* **6**, 1601167 (2017).

9 Molina-Lopez, F. *et al.* Inkjet-printed stretchable and low voltage synaptic transistor array. *Nature Communications* **10**, 1-10 (2019).

10 Garnier, F., Hajlaoui, R., Yassar, A. & Srivastava, P. All-polymer field-effect transistor realized by printing techniques. *Science* **265**, 1684-1686 (1994).

11 Shaheen, S. E., Radspinner, R., Peyghambarian, N. & Jabbour, G. E. Fabrication of bulk heterojunction plastic solar cells by screen printing. *Applied Physics Letters* **79**, 2996-2998 (2001).

12 Ersman, P. A. *et al.* All-printed large-scale integrated circuits based on organic electrochemical transistors. *Nature Communications* **10**, 1-9 (2019).

13 Sessolo, M. *et al.* Easy-to-fabricate conducting polymer microelectrode arrays. *Advanced Materials* **25**, 2135-2139 (2013).

14 Wang, S. *et al.* Skin electronics from scalable fabrication of an intrinsically stretchable transistor array. *Nature* **555**, 83 (2018).

15 Liu, Y. *et al.* Soft conductive micropillar electrode arrays for biologically relevant electrophysiological recording. *Proceedings of the National Academy of Sciences* **115**, 11718-11723 (2018).

16 Liu, Y. *et al.* Soft and elastic hydrogel-based microelectronics for localized low-voltage neuromodulation. *Nature Biomedical Engineering* **3**, 58 (2019).

17 Sekine, S., Ido, Y., Miyake, T., Nagamine, K. & Nishizawa, M. Conducting polymer electrodes printed on hydrogel. *Journal of the American Chemical Society* **132**, 13174-13175 (2010).

18 Ido, Y. *et al.* Conducting polymer microelectrodes anchored to hydrogel films. *ACS Macro Letters* **1**, 400-403 (2012).
19 Feig, V. R. *et al.* An electrochemical gelation method for patterning conductive PEDOT: PSS hydrogels. *Advanced Materials* **31**, 1902869 (2019).
20 Singh, R., Tharion, J., Murugan, S. & Kumar, A. ITO-free solution-processed flexible electrochromic devices based on PEDOT: PSS as transparent conducting electrode. *ACS Applied Materials & Interfaces* **9**, 19427-19435 (2016).
21 Fan, X., Wang, J., Wang, H., Liu, X. & Wang, H. Bendable ITO-free organic solar cells with highly conductive and flexible PEDOT: PSS electrodes on plastic substrates. *ACS Applied Materials & Interfaces* **7**, 16287-16295 (2015).
22 Ouyang, J. Solution-processed PEDOT: PSS films with conductivities as indium tin oxide through a treatment with mild and weak organic acids. *ACS Applied Materials & Interfaces* **5**, 13082-13088 (2013).
23 Yao, B. *et al.* Ultrahigh-conductivity polymer hydrogels with arbitrary structures. *Advanced Materials* **29**, 1700974 (2017).
24 Feig, V. R., Tran, H., Lee, M. & Bao, Z. Mechanically tunable conductive interpenetrating network hydrogels that mimic the elastic moduli of biological tissue. *Nature Communications* **9**, 2740 (2018).
25 Lu, B. *et al.* Pure PEDOT:PSS hydrogels. *Nature Communications* **10**, 1043 (2019).
