# Supplementary material for: 3D printing of conducting polymers
Source: Nat Commun. 2020 Mar 30;11:1604. doi: 10.1038/s41467-020-15316-7 (PMC7105462; doi:10.1038/s41467-020-15316-7)
Supplement: Supplementary file 2 — Description of Additional Supplementary Files [file 41467_2020_15316_MOESM2_ESM.pdf]

Description of Additional Supplementary Files

File Name: Supplementary Movie 1
Description: 3D printing of a 20-layered high aspect ratio structure by the conducting polymer ink.

File Name: Supplementary Movie 2
Description: 3D printing of overhanging features over high aspect ratio structures by the conducting polymer ink.

File Name: Supplementary Movie 3
Description: 3D printing of a multi-electrodes array (MEA) by the conducting polymer ink and PDMS ink.

File Name: Supplementary Movie 4
Description: High resolution and high throughput 3D printing of flexible circuit patterns by the conducting polymer ink.

File Name: Supplementary Movie 5
Description: 3D printing of a soft multi-channel neural probe by the conducting polymer ink and PDMS ink.
